# Lipid Accumulation Product Is More Related to Insulin Resistance than the Visceral Adiposity Index in the Maracaibo City Population, Venezuela

**DOI:** 10.1155/2021/5514901

**Published:** 2021-06-07

**Authors:** Valmore Bermúdez, Juan Salazar, Jorge Fuenmayor, Manuel Nava, Ángel Ortega, Pablo Duran, Milagros Rojas, Roberto Añez, Alejandra Rivas-Montenegro, Lissé Angarita, Maricarmen Chacín, Clímaco Cano, Manuel Velasco, Joselyn Rojas

**Affiliations:** ^1^Universidad Simón Bolívar, Facultad de Ciencias de La Salud, Barranquilla, Colombia; ^2^Endocrine and Metabolic Diseases Research Center, School of Medicine, University of Zulia, Maracaibo, Venezuela; ^3^Department of Endocrine and Nutrition, Gregorio Marañón General University Hospital, Madrid, Spain; ^4^Universidad Andres Bello, Carrera de Nutrición, Concepción, Chile; ^5^Universidad Central de Venezuela, Escuela de Medicina José María Vargas, Caracas, Venezuela; ^6^Division of Pulmonary and Critical Care Medicine, Brigham and Women's Hospital and Harvard Medical School, Boston, MA 02115, USA

## Abstract

**Background:**

Visceral adiposity is related to insulin resistance (IR), a metabolic state considered as a risk factor for other cardiometabolic diseases. In that matter, mathematical indexes such as the visceral adiposity index (VAI) and the lipid accumulation product (LAP) could indirectly assess IR based on visceral adiposity.

**Objective:**

To evaluate the association and diagnostic accuracy of VAI and LAP to diagnose IR in the adult population of Maracaibo city.

**Methods:**

This is a cross-sectional descriptive study with multistage sampling. Receiver operating characteristic (ROC) curves were built to determine VAI and LAP cutoff points to predict IR. A set of logistic regression models was constructed according to sociodemographic, psychobiologic, and metabolic variables.

**Results:**

1818 subjects were evaluated (51.4% women). The area under the curve (AUC) values for LAP and VAI were 0.689 (0.665–0.714) and 0.645 (0.619–0.670), respectively. Both indexes showed a higher IR risk in the upper tertile in bivariate analysis. However, in the logistic regression analysis for the IR risk, only the 2nd (OR: 1.91; 95% CI: 1.37–2.65; *p* < 0.01) and 3rd (OR: 5.40; 95% CI: 3.48–8.39; *p* < 0.01) LAP tertiles showed a significant increase. This behaviour was also observed after adjusting for hs-C-reactive protein (hs-CPR).

**Conclusion:**

Although both indexes show a low predictive capacity in individuals with IR in the Maracaibo city population, the LAP index was more strongly associated with IR.

## 1. Introduction

Insulin resistance (IR) is a metabolic state in which insulin-dependent tissues progressively lose their sensitivity to this hormone. This phenomenon leads to metabolic imbalance and hyperinsulinemia [1]. Measuring IR precisely allows for the identification of individuals at risk of developing pathologies associated with this metabolic scenario, such as metabolic syndrome (MS), type 2 diabetes mellitus (T2DM), or polycystic ovarian syndrome (PCOS). Therefore, it allows for the implementation of timely and specific interventions [2]. The hyperinsulinemic-euglycemic clamp is considered the gold standard for the determination of IR. However, it is costly, and it requires time and trained personnel, especially for large-scale epidemiologic studies [3]. For this reason, more practical and accessible alternatives have been proposed, such as the homeostatic model assessment (HOMA) to evaluate IR, the quantitative insulin check index (QUICKI), and the Matsuda index for clinical use. Meanwhile, McAuley, Avignon, and Stumvoll indexes are more appropriate for epidemiologic and research studies [4].

The visceral adiposity index (VAI) represents an empiric sex-specific model proposed as a substitute marker of dysfunction and distribution of adipose tissue and its independent correlation with cardiometabolic risk. Recent research has determined a close relationship between the use of VAI and peripheral glycemia, IR, and T2DM [5, 6]. On the contrary, the lipid accumulation product (LAP) expresses a constant adipose risk function associated with the development of cardiovascular disease and mortality in adults. It exhibits a predictive capacity to identify subjects at risk of presenting cardiovascular events as the age increased, which was observed more strongly in men [7]. Both indexes have been initially proposed as simple and easy tests to evaluate visceral adipose tissue dysfunction and cardiometabolic risk [8].

Furthermore, these indexes have proven to be valuable tools in identifying prediabetes, T2DM [9], and MS in patients with chronic kidney disease. Noticeably, LAP is superior in both women and men [10]. On the contrary, both VAI and LAP have proven to be promising markers to identify IR and cardiometabolic risk in obese and nonobese women with PCOS [11]. There is no consensus regarding the clinical index that shows a better discriminatory capacity to determine IR and cardiometabolic risk. Therefore, this study aimed to evaluate the association and diagnostic accuracy of VAI and LAP in diagnosing IR in the adult population from Maracaibo city.

## 2. Materials and Methods

### 2.1. Study Design and Selection of Subjects

The Maracaibo City Metabolic Syndrome Prevalence Study (MMSPS) was a cross-sectional, descriptive study performed in Maracaibo city, Venezuela. The methodology and main results of the study have been previously reported [12, 13]. The subjects without insulin determination, type 2 and type 1 diabetes mellitus patients, and polycystic ovary syndrome patients were excluded in this secondary analysis; therefore, a total of 1818 subjects were evaluated. This study was approved by the Endocrine and Metabolic Diseases Research Center's Bioethics Committee (approval number: BEC-006-0305). All participants signed a written informed consent form before being interviewed and examined by a trained team.

### 2.2. Clinical Evaluation

All subjects included in the study went through a medical examination performed by trained personnel to obtain a complete clinical record. During the anamnesis, information about the past medical and family history of endocrine and metabolic disorders was collected. This record included age, race, working status, education, and socioeconomic status using the Graffar scale modified by Mendez-Castellano to evaluate the last aspect [14].

Subjects were asked about smoking habits and their duration, and they were categorized as current smokers, former smokers, and nonsmokers [15]. Physical activity was evaluated by the International Physical Activity Questionnaire [16]. For alcohol consumption, any subject that drank ≥1 gram daily was considered as a “drinker.” [17]

Height was obtained using a calibrated rod in millimeters and centimeters, with the patient barefooted and his/her back facing the wall. Weight was recorded using a digital scale (Tanita, TBF-310 GS Body Composition Analyzer, Tokyo, Japan) with the patient wearing light clothing and no shoes. Body mass index (BMI) was calculated by applying Quetelet's equation (weight/height^2^). The subjects were classified according to the following: underweight below 18.50 kg/m^2^, normal weight between 18.50 and 24.99 kg/m^2^, overweight (preobese) between 25.00 and 29.99 kg/m^2^, obese class I between 30.00 and 34.99 kg/m^2^, obese class II between 35.00 and 39.99 kg/m^2^, and obese class III beyond 40.00 kg/m^2^. WC was measured using calibrated measuring tapes in millimeters and centimeters, using anatomical landmarks according to the National Institutes of Health protocol: the midpoint between the lower border of the rib cage and the iliac crest, measuring at the end of expiration with participants standing and wearing only their undergarments [18]. Finally, blood pressure was determined by the auscultatory method using an adequately calibrated and validated sphygmomanometer (Tycos DS48, Welch Allyn). High blood pressure was defined according to the cutoff points proposed for metabolic syndrome in the 2009 IDF/AHA/NHLBI/WHF/IAS/IASO consensus (≥130/85 mmHg) [19].

### 2.3. Laboratory Test Panel

After 8 hours of fasting, a blood sample was taken from the cubital vein and then centrifuged to obtain serum. Serum levels of glucose, total cholesterol (TC), and triacylglycerides (TAG) were determined using enzymatic colorimetric kits (HUMAN Gesellshaft Biochemica and Diagnostica mbH) and a specialized computer system. The intra-assay variation coefficients for TC, TAG, and high-density lipoprotein cholesterol (HDL-C) were 3%, 5%, and 5%, respectively. Insulin was determined by a double-sandwich ultrasensitive ELISA commercial kit (DRG Instruments GmbH, Germany, Inc.). HOMA2-IR was used for IR evaluation as proposed by Levy et al. [20], and its calculation was done with the HOMA Calculator, version 2.2.2. IR was defined by a HOMA2-IR cutoff point of ≥2 [21]. Serum high-sensitivity C-reactive protein (hs-CRP) levels were quantified using a commercial immunoturbidimetric assay (Human Gesellshaft Biochemica and Diagnostica mbH), setting the cutoff point at ≥0.765 mg/L [22].

### 2.4. Adiposity Indexes

VAI calculation was performed with the gender-specific equations proposed by Amato et al. [23] (Supplementary formula 1). LAP calculation was made applying the equation previously published by Kahn [24] (Supplementary formula 2).

### 2.5. Statistical Analysis

Qualitative variables were expressed in absolute and relative frequencies, and the possible relationship between these variables was assessed with the *χ*^2^ test (chi-square). The difference in proportions was determined through a *Z*-test. Quantitative variables were expressed in means ± standard deviation when there was a normal distribution and medians (interquartile range) when they had a nonnormal distribution. Student's *t*-test and Mann–Whitney U test were used for normal and nonnormal distributed variables to assess arithmetic mean differences between two groups, respectively.

Receiver operating characteristic (ROC) curves were constructed to set the appropriate IR cutoff point in both the general population and according to gender for VAI and LAP. The curves were plotted using the package pROC running in R Project software for statistical computing, optimal cutoff points were selected using the Youden index, and the distance closest to ROC and differences between two or more AUCs were assessed through DeLong's test [25].

Multiple logistic regression models were constructed (95% confidence intervals) for IR presence and adjusted by age, ethnic group, education level, socioeconomic status, working status, diabetes mellitus, family history, alcohol intake, smoking habits, leisure-domain physical activity, and high blood pressure according to the IDF/AHA/NHLBI/WHF/IAS/IASO 2009 consensus criteria. The second model adds hs-CRP. Data were analyzed using Statistical Package for the Social Sciences (SPSS) v.23 for Windows (IBM, Chicago, IL), and statistically significant results were determined when *p* < 0.05.

## 3. Results

### 3.1. General Characteristics of the Sample

Out of the 1818 individuals studied, 51.4% (*n* = 934) were women. The overall arithmetic mean for age was 37.7 ± 13.9 years, and the largest age group was the 30–59-year-old group (55.1%; *n* = 1001). It was observed that 43.3% of the participants had IR.  Table 1 depicts the general features of the studied sample.

### 3.2. Adiposity Indexes and Insulin Resistance Prediction

**Figure 1:**
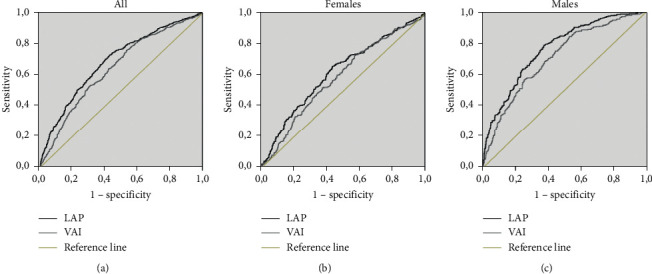
ROC curves of LAP and VAI for insulin resistance (Maracaibo, Venezuela). LAP: lipid accumulation product; VAI: visceral adiposity index.

The LAP and VAI ROC for IR prediction in both the general population and the population distributed according to sex are shown in Figure 1. Overall, the AUC values for LAP and VAI were 0.689 (0.665–0.714) and 0.645 (0.619–0.670), respectively. On the contrary, the AUC values for LAP and VAI for women were 0.621 (0.584–0.657) and 0.587 (0.550–0.624), respectively, whereas in men, the values were 0.759 (0.728–0.791) for LAP and 0.704 (0.670–0.738) for VAI. The sensitivity, specificity, Youden index, and distance to ROC are depicted in Table 2.

### 3.3. Adiposity Indexes and Insulin Resistance Association

Table 3 shows the degree of association between both adiposity indexes and IR. In the univariate analysis, the subjects located on the upper LAP tertile (>51.2) showed a higher IR frequency (50.2%; *n* = 394). Likewise, the subjects in the upper VAI tertile (>2.2) have higher IR frequency (45.3%; *n* = 357). However, in the logistic regression models, only the LAP tertiles showed a significant increase of IR risk (tertile 2: OR: 1.91; 95% CI: 1.37–2.65; *p* < 0.010; tertile 3: OR: 5.40; 95% CI: 3.48–8.39; *p* < 0.01). This relationship remained without change after hs-CRP adjustment.

## 4. Discussion

Numerous investigations have determined that mathematical indexes such as VAI and LAP allow for the indirect evaluation of IR from visceral adiposity [6, 26, 27]. In the present study, the association and diagnostic precision of VAI and LAP in identifying IR in adults from Maracaibo city were evaluated. AUC values were more significant for LAP than for VAI. Regarding multivariate analysis, LAP proved to be a better predictor for IR presence than VAI, and therefore an index with a better discriminative power to assess IR in this population.

Since visceral adiposity is related to the development of metabolic pathologies, VAI and LAP indexes have been used as indirect and more economical predictors of T2DM, prediabetes [28], IR [29], or MS [30]. However, among the variables used to determine the visceral adiposity index, there are MS diagnosis criteria. Therefore, a correlation between them could exist [27, 31], and they should not be used to diagnose SM. Likewise, other authors have used VAI and LAP to establish the presence of T2DM and prediabetes [32]. In this study, we decided to evaluate a premorbid condition such as IR, which tends to be associated with visceral adiposity and its dysfunction due to the convergence of molecular mechanisms in both metabolic disorders.

According to the ROC analysis, it was shown that LAP is the index with the greater IR predictive capacity in the general population and sex. Similar to these findings, other studies have also compared different IR surrogate indexes, reporting that the LAP index has better discrimination for the identification of IR. Fiorentino et al. performed a study of 631 individuals with different degrees of glucose intolerance who were part of the European Network on Functional Genomics of Type 2 Diabetes (EUGENE2) Project from Italy. After the ROC analysis, these researchers found that the capacity of the LAP index to identify individuals with IR defined by the AUC value (0.728; 95% CI: 0.692–0.762) was higher than VAI (0.688; 95% CI: 0.650–0.724), TAG/HDL-C ratio (0.693; 95% CI: 0.646–0.729), and TyG index (0.688; 95% CI: 0.650–0.723) (*p* < 0.05) [33]. Likewise, in another study performed by Mazidi et al. in 18,318 adult individuals from the NHANES data, it was found that the LAP index had a higher IR prediction capacity (0.810; 95% CI: 0.788–0.831) when compared to other traditional tools such as VAI (0.750; 95% CI: 0.727–0.775) [34]. However, differing from the findings in our study, other researchers have reported that AUC levels for LAP and VAI were similar [11, 35–37], suggesting that both indexes have the same clinical usefulness in identifying insulin-resistant individuals. These differences in the predictive role of the visceral adiposity indexes among population groups could be due to the evolution and natural history of obesity in the subjects. Other factors involved would be the time of interaction with other cardiometabolic risk factors, the differences in the frequency of these risk factors in different populations, and the influence of ethnicity and genetic considerations.

Alternatively, although higher LAP and VAI levels were associated with a higher IR frequency, only high LAP levels showed a significantly higher risk of presenting IR. These findings were similar to those reported by Oh et al., who demonstrated that high LAP levels were associated with higher HOMA-IR levels in healthy Korean women [38]. Likewise, a cross-sectional study, including 2,524 nondiabetic Chinese subjects, found that HOMA-IR scores significantly rose as LAP increased [39]. Different authors have also shown a higher positive correlation between LAP and HOMA-IR compared to VAI [33, 37, 39]. In this sense, Mazidi et al. reported a significantly higher positive correlation between HOMA-IR and LAP (*r* = 0.551) compared to other indexes such as the TyG index (*r* = 0.502), apVAT (*r* = 0.454), TAG : HDL-C ratio (*r* = 0.441), and VAI (*r* = 0.451) [34]. These results were similar to those reported by Xia et al. in which HOMA-IR was positively correlated to LAP (*r* = 0.361), WC (*r* = 0.347), and IMC (*r* = 0.346). In the same study, through a multivariant logistic regression, it was shown that LAP has a higher impact on HOMA-IR scores compared to BMI in men (standardized coefficient *β* = 0.22, *p* < 0.001 vs. standardized coefficient *β* = 0.20, *p* < 0.001) and women (standardized coefficient *β* = 0.24, *p* < 0.001 vs. standardized coefficient *β* = 0.15, *p* < 0.001) [37]. However, VAI was not included in this analysis. Similarly, other studies have highlighted the superiority of LAP in contrast to other indexes used in individuals with T2DM [26], MS, and cardiovascular disease [40] and women with PCOS [29].

These findings could be explained since both consider WC as an anthropometric parameter of central obesity. They also include concentrations of proatherogenic lipids (TAG in the case of LAP) and antiatherogenic lipids (HDL-C in the case of VAI), which together would allow for the evaluation of adiposity status and to indirectly estimate the metabolic profile of the individuals [41]. IR leads to hypertriglyceridemia due to the inability of insulin to inhibit lipid production at the hepatic level [42, 43]. Furthermore, hypertriglyceridemia has been associated with the ectopic deposit and accumulation of lipids in visceral tissue [44, 45]. Meanwhile, although WC does not distinguish between subcutaneous and visceral tissue, it has been strongly associated with higher cardiometabolic risk [46]. In this sense, visceral adipose tissue has a higher rate of lipolysis and proinflammatory adipokines' secretion, promoting a low-grade inflammation state, which alters insulin signalling [47–49]. By including parameters associated with metabolic disturbances associated with deflective insulin action, LAP positions itself as a better IR predictor [50].

Our research has some limitations, among which it is essential to highlight the cross-sectional nature of the study, which avoids the establishment of relationships of causality among the indexes. Additionally, instead of using the gold standard in evaluating IR (hyperinsulinemic-euglycemic clamp technique), HOMA2-IR was used, which is a tool validated in different populations, especially in epidemiologic analyses in low-resource contexts [50].

In conclusion, LAP shows a higher predictive capacity and association with IR in the Maracaibo city population than VAI and could be an easy and nonexpensive tool that can be used in low-income settings.

## Figures and Tables

**Table 1:** General characteristics of the sample (Maracaibo, Venezuela).

	Without IR (*n* = 1031)		With IR (*n* = 787)		Total (*n* = 1818)	
	*n*	(%)	*n*	(%)	*n*	(%)
Gender						
Female	531	51.5	403	51.2	934	51.4
Male	500	48.5	384	48.8	884	48.6

Age (years)						
<30	394	38.2	259	32.9	653	35.9
30–59	542	52.6	459	58.3	1001	55.1
≥60	95	9.2	69	8.8	164	9.0


BMI (kg/m^2^) (mean ± SD)^*∗*^	26.3 ± 5.2		30.3 ± 6.4		28.0 ± 6.1	
WC (cm) (mean ± SD)^*∗*^	89.7 ± 12.6		99.4 ± 15.9		93.9 ± 14.9	
Triglycerides (mmol/L) (median *p*25–*p*75th)^*∗∗*^	1 (0.7–1.5)		1.3 (0.9–1.9)		1.2 (0.8–1.7)	
HDL-C (mmol/L) (median *p*25–*p*75th)^*∗∗*^	1.1 (0.9–1.4)		1.1 (0.9–1.2)		1.1 (0.9–1.3)	
LAP (median *p*25–*p*75th)^*∗∗*^	27.8 (14.7–49.1)		51.32 (30.1–84.5)		36.1 (18.7–63.5)	
VAI (median *p*25–*p*75th)^*∗∗*^	1.4 (0.9–2.3)		2.1 (1.3–3.2)		1.7 (1–2.7)	

IR: insulin resistance; BMI: body mass index; SD: standard deviation; WC: waist circumference; LAP: lipid accumulation product; VAI: visceral adiposity index. ^*∗*^Student's *t*-test, *p* < 0.01. ^*∗∗*^Mann–Whitney U test, *p* < 0.01.

**Table 2:** Diagnostic parameters of LAP and VAI for insulin resistance prediction (Maracaibo, Venezuela).

	All		Females		Males	
	LAP	VAI	LAP	VAI	LAP	VAI
Cutoff points	37.7	1.7	33.4	1.6	41.7	1.7
AUC (95% CI)	0.689 (0.665–0.714)	0.645 (0.619–0.670)	0.621 (0.584–0.657)	0.587 (0.550–0.624)	0.759 (0.728–0.791)	0.704 (0.670–0.738)
Sensitivity (%)	64.7	60.5	60.3	60.8	70.1	66.4
Specificity (%)	64.3	60.1	60.3	52.5	68.0	63.0
Youden index	0.29	0.39	0.21	0.13	0.38	0.29
Distance to ROC	0.50	0.56	0.56	0.62	0.44	0.49

LAP: lipid accumulation product; VAI: visceral adiposity index. Delong's test between gender: *p* < 0.05.

**Table 3:** Association between adiposity indexes and insulin resistance (Maracaibo, Venezuela).

	Without IR		With IR		OR (95% CI); *p*^*∗*^	OR (95% CI); *p*^*∗∗*^
	*n*	(%)	*n*	(%)		
*Lipid accumulation product*						
<23, 6	444	43, 1	152	19, 3	1.00	1.00
23, 6–51, 2	354	34, 3	241	30, 6	1.91 (1.37–2.65); <0.001	1.91 (1.27–2.88); 0.002
>51, 2	233	22, 6	39	50, 1	5.40 (3.48–8.39); <0.001	6.03 (3.45–1.52); <0.001

*Visceral adiposity index*						
<1, 2	430	41, 7	158	20, 1	1.00	1.00
1,2–2, 2	337	32, 7	272	34, 6	1.34 (0.98–1.84); 0.07	1.35 (0.91–1.99); 0.14
>2, 2	264	25, 6	357	45, 3	1.14 (0.76–1.72); 0.53	0.98 (0.58–1.65); 0.94

Chi-square test: LAP (176.3; *p* < 0.01); VAI (116.0; *p* < 0.01). ^*∗*^Adjusted models for gender, age, ethnic group, education level, socioeconomic status, working status, diabetes mellitus family history, alcohol intake, smoking habits, leisure-domain physical activity, and high blood pressure according to the IDF/AHA/NHLBI/WHF/IAS/IASO 2009 consensus criteria. ^*∗∗*^Adjusted similar to model 1 plus hs-C-reactive protein.

## Data Availability

The excel template data used to support the findings of this study have been deposited in the F1000 research repository (10.5256/f1000research.14571.d201851).

## Supplementary Materials

Supplementary Materials Supplementary formula 1: visceral adiposity index formula. TAG and HDL-C are expressed in mmol/L. VAI: visceral adiposity index; WC: waist circumference; BMI: body mass index; TAG: triacylglycerides; HDL-C: high-density lipoprotein cholesterol. Supplementary formula 2: lipid accumulation product formula. TAG are expressed in mmol/L. LAP: lipid accumulation product; WC: waist circumference; TAG: triacylglycerides.
