# Supplementary material for: Lipid Accumulation Product Is More Related to Insulin Resistance than the Visceral Adiposity Index in the Maracaibo City Population, Venezuela
Source: J Obes. 2021 Jun 7;2021:5514901. doi: 10.1155/2021/5514901 (PMC8203405; doi:10.1155/2021/5514901)

**Supplementary** **Formula 1.** Visceral Adiposity Index formula. TAG and HDL-C are expressed in mmol/L. Abbreviations: VAI: Visceral Adiposity Index; WC: Waist Circumference; BMI: Body Mass Index; TAG: Triacylglycerides; HDL-C: High Density Lipoprotein Cholesterol.


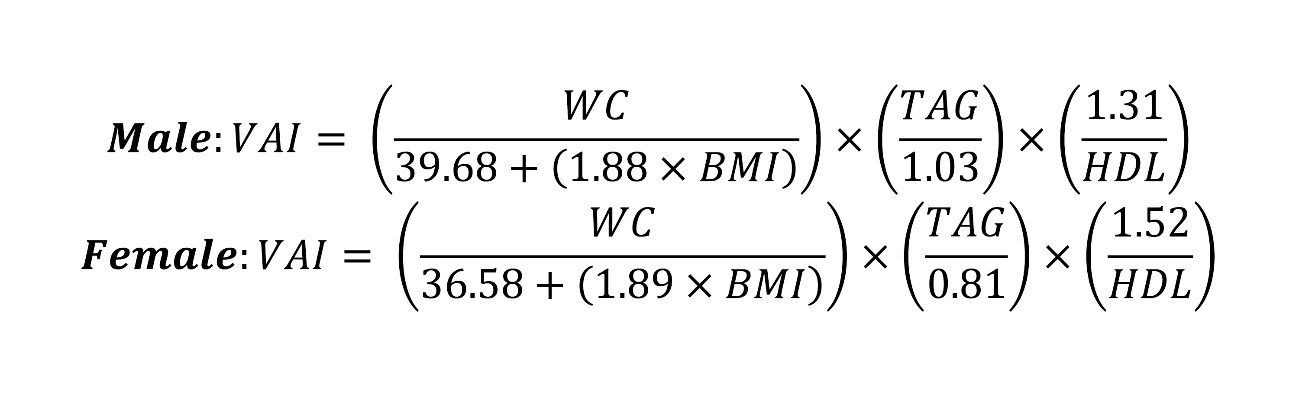


**Supplementary** **Formula 2**. Lipid Accumulation Product formula. TAG are expressed in mmol/L. Abbreviations: LAP: Lipid Accumulation Product; WC: Waist Circumference; TAG: Triacylglycerides.


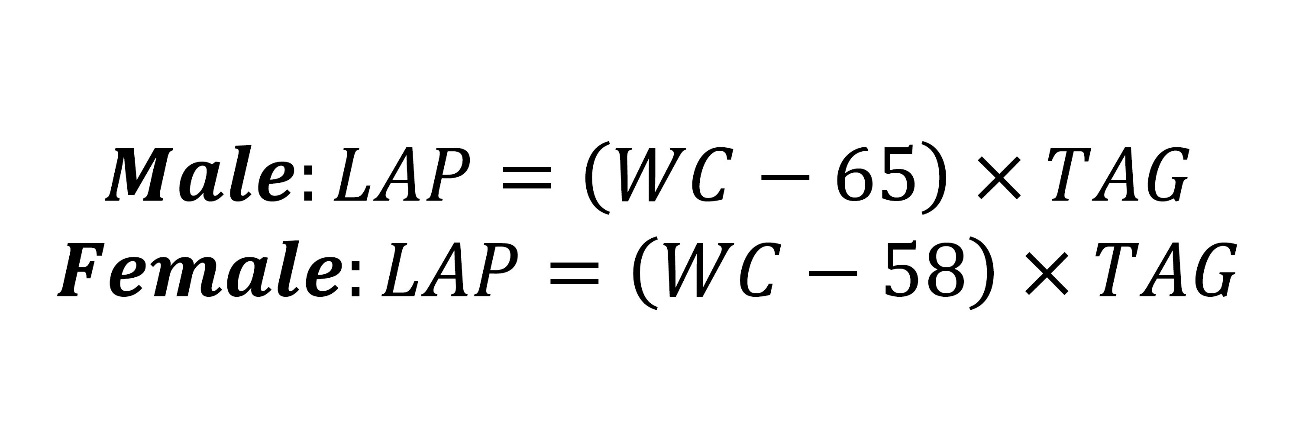

Supplement: Supplementary Materials — Supplementary formula 1: visceral adiposity index formula. TAG and HDL-C are expressed in mmol/L. VAI: visceral adiposity index; WC: waist circumference; BMI: body mass index; TAG: triacylglycerides; HDL-C: high-density lipoprotein cholesterol. Supplementary formula 2: lipid accumulation product formula. TAG are expressed in mmol/L. LAP: lipid accumulation product; WC: waist circumference; TAG: triacylglycerides. [file 5514901.f1.docx]
